# Diastolic Dysfunction Unveiling Cardiac Light-Chain Amyloidosis: A Case Report

**DOI:** 10.18103/mra.v12i12.6168

**Published:** 2024-12

**Authors:** Somesh Saha, Ritwick Mondal, Shramana Deb, Biswarup Sarkar, Julián Benito-León

**Affiliations:** 1 Department of Critical Care Medicine, Belle Vue Clinic, Kolkata, India.; 2 Department of Clinical Pharmacology and Therapeutic Medicine, IPGMER and SSKM Hospital, Kolkata, India.; 3 Department of Stroke Medicine, Institute of Neurosciences, Kolkata, India.; 4 Department of Cardiology, Medical College, Kolkata, India.; 5 Department of Neurology, University Hospital “12 de Octubre”, Madrid, Spain.; 6 Instituto de Investigación Sanitaria Hospital 12 de Octubre (imas12), Madrid, Spain.; 7 Centro de Investigación Biomédica en Red Sobre Enfermedades Neurodegenerativas (CIBERNED) Madrid, Spain.; 8 Department of Medicine, Complutense University, Madrid, Spain.

**Keywords:** Light chain, amyloidosis, cardiac amyloidosis, hypertrophy, Heart failure

## Abstract

**Background:**

Cardiac light-chain amyloidosis represents a critical component of this multi-systemic disease, significantly impacting prognosis. The extent of cardiac free light-chain deposition is the primary determinant of survival.

**Case Presentation:**

We report the case of a 67-year-old male with a 10-year history of diabetes mellitus and arterial hypertension who presented with a two-day history of chest discomfort and difficulty lying down or sleeping, along with a two-month history of progressively worsening exertional dyspnea. On examination, the patient exhibited low blood pressure. A 12-lead electrocardiogram revealed poor R-wave progression and left ventricular hypertrophy. Further evaluation using 2D echocardiography demonstrated significant concentric left ventricular hypertrophy, a restrictive filling pattern, and mild pericardial effusion. Cardiac magnetic resonance imaging, nuclear imaging, and biopsy confirmed the diagnosis of cardiac light-chain amyloidosis.

**Conclusion:**

Timely recognition and a high index of suspicion are essential for the early diagnosis of cardiac amyloidosis. Prompt diagnosis enables the initiation of definitive therapy, which may halt disease progression and significantly improve prognosis.

## Background

Cardiac amyloidosis has garnered increasing attention over the past decades, as cardiac involvement is now recognized as a leading cause of mortality in this multi-system disease.^1,2^ Extracellular deposition of insoluble β-sheet fibrillar proteins in the heart has been identified in 30 different types of amyloidosis.^3^ Among these, immunoglobulin-derived light chains and transthyretin are the most common culprits, causing amyloid light-chain and transthyretin cardiac amyloidosis, respectively. Amyloid light-chain amyloidosis is the more prevalent subtype.^4^

Cardiac light-chain amyloidosis typically presents with symptoms such as heart failure, hypotension, and atrial or ventricular arrhythmias and is associated with a poor prognosis, with a median survival of only 6–9 months. Here, we describe a 67-year-old male with newly diagnosed cardiac light-chain amyloidosis, whose case highlights the critical role of a multidisciplinary approach in guiding diagnosis and management strategies.

## Case Presentation

A 67-year-old male with a 10-year history of type 2 diabetes mellitus and arterial hypertension was admitted to the emergency department with complaints of chest discomfort and difficulty lying down and sleeping for the past two days. He reported a two-month history of progressive exertional dyspnea and fatigue. His medical history included a prior hospitalization for COVID-19. On initial evaluation, his vital signs were as follows: blood pressure, 100/70 mmHg; pulse, 81 bpm; respiratory rate, 16/min; and SpO_2_, 97% on room air. Auscultation revealed bilateral basal crackles, and a cardiovascular examination was noted to show normal S1 and S2 sounds.

**Figure 1:**
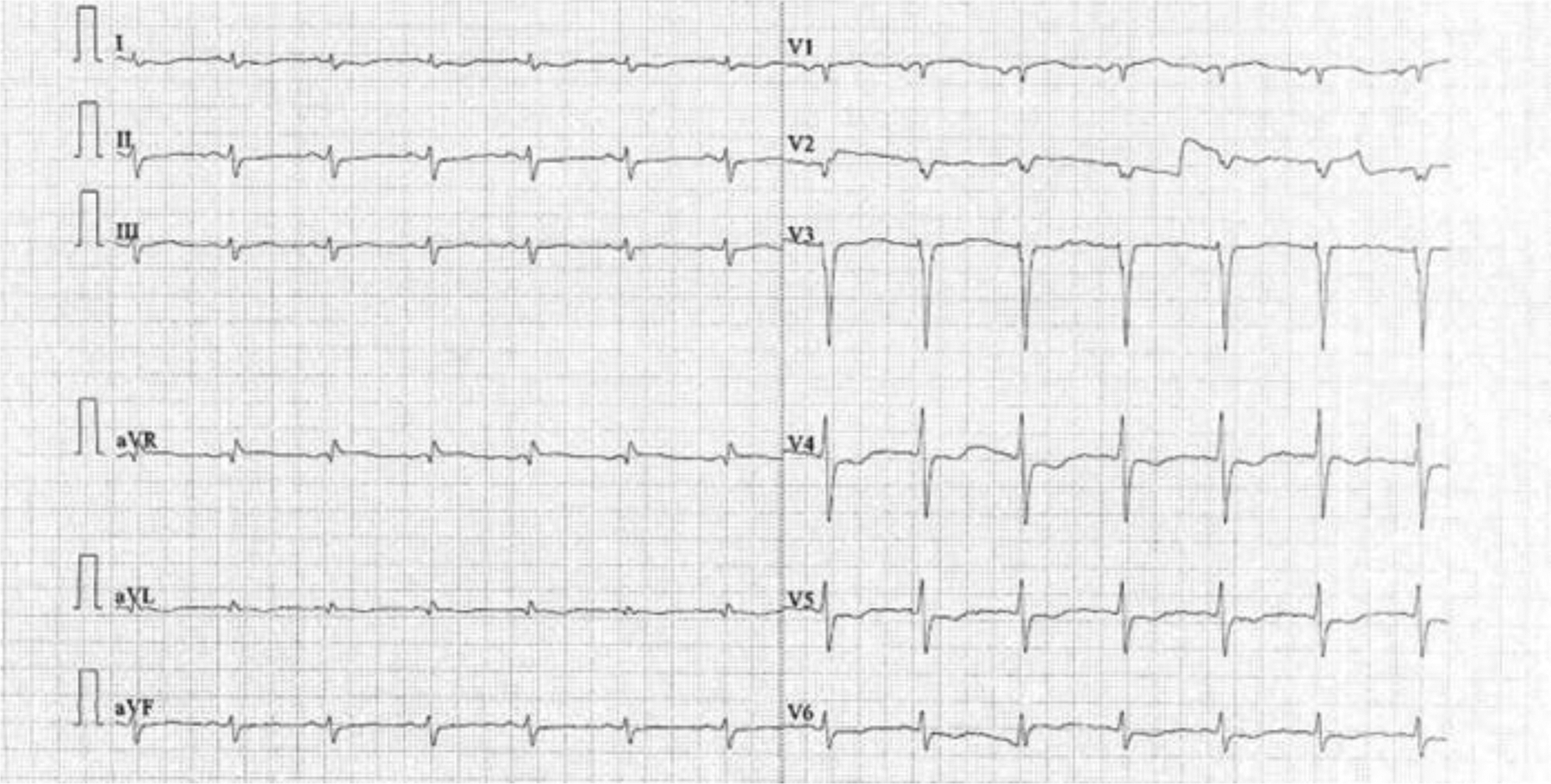
Poor R-wave progression in V1-V4 chest leads with left ventricular hypertrophy on 12-lead electrocardiogram.

12-lead ECG revealed abnormal Q waves, poor R-wave progression, and evidence of left ventricular hypertrophy, accompanied by T-wave inversions in leads I, aVL, V4, V5, and V6 [Figure 1]. Laboratory results revealed elevated NT-proBNP (5398 pg/mL) and Troponin-T (95.40 pg/mL). COVID-19 RT-PCR was negative. The patient was initiated on low-dose aspirin, bisoprolol (2.5 mg/day), and statin therapy before referral to the cardiac intensive care unit.

**Figure 2:**
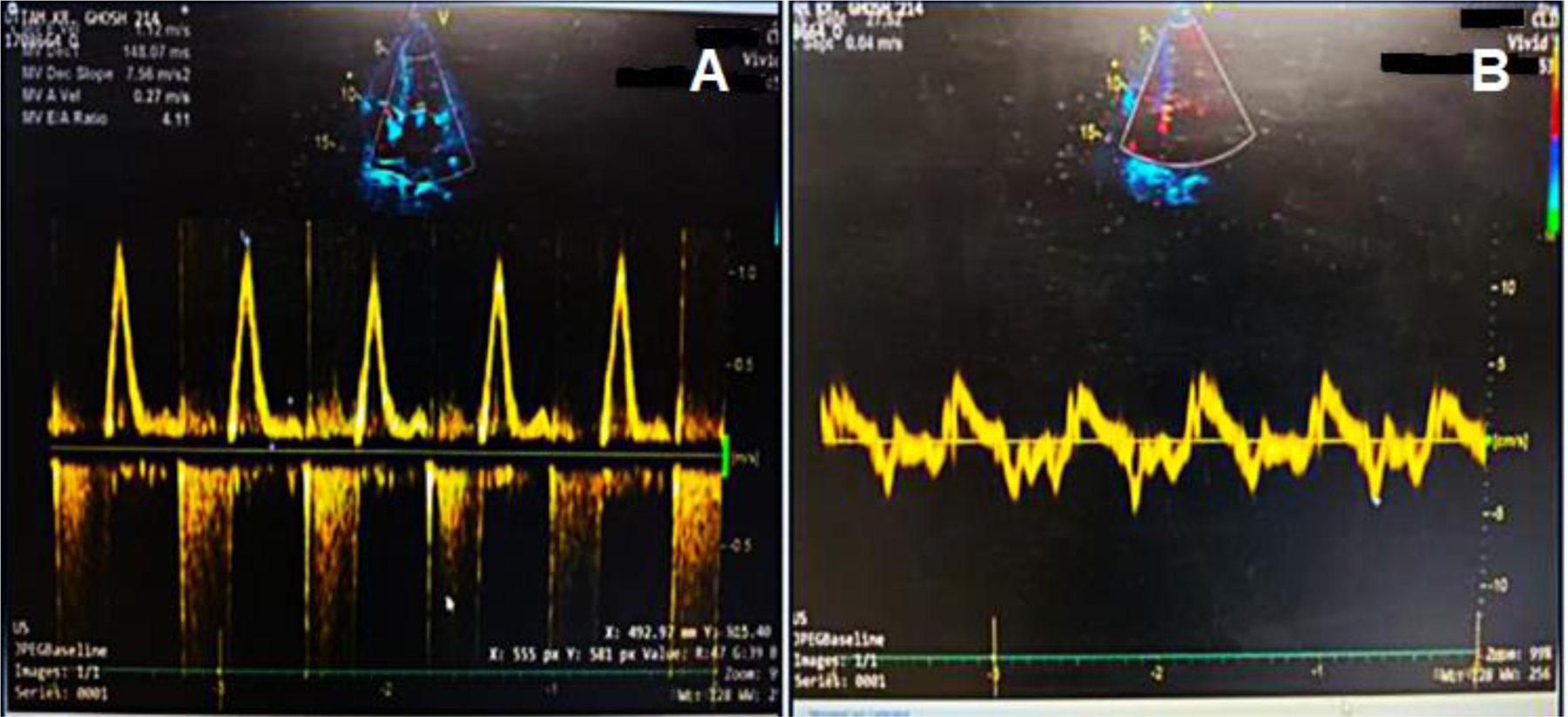
(a) Marked increase in early diastolic mitral inflow velocity with reduced late diastolic peak flow velocity and deceleration. (b) Decreased early diastolic mitral annulus velocity is shown.

Transthoracic echocardiography revealed significant concentric left ventricular hypertrophy, a restrictive filling pattern (E/e’: 29.4, DT: 148 ms, E/A: 4.1), and mild pericardial effusion (posteriorly: 6 mm, anteriorly: 4 mm) [Figure 2(a), 2(b)]. Additional findings included a left ventricular ejection fraction of 60%, enlarged atria, normal right ventricular function (tricuspid annular plane systolic excursion: 18 mm), moderate mitral and tricuspid regurgitation, trivial aortic regurgitation, and moderate pulmonary artery hypertension (pulmonary artery systolic pressure: 60 mmHg). Differential diagnoses included restrictive cardiomyopathy, restrictive pericarditis, valvular heart disease, acute coronary syndrome, ischemic heart disease, hypertensive cardiomyopathy, and post-COVID sequelae.

**Figure 3:**
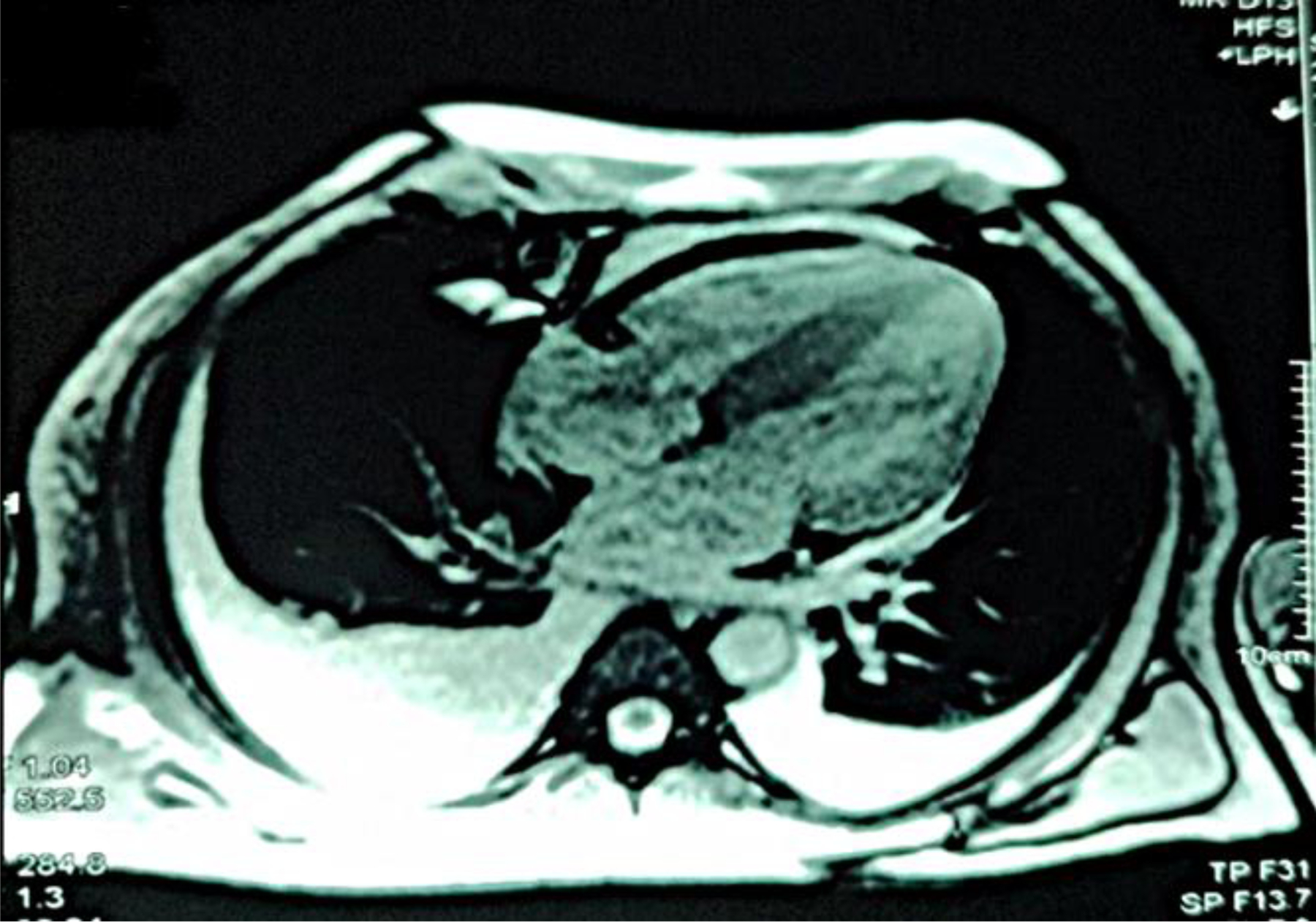
Diffuse global late gadolinium enhancement observed on cardiac magnetic resonance imaging.

**Figure 4:**
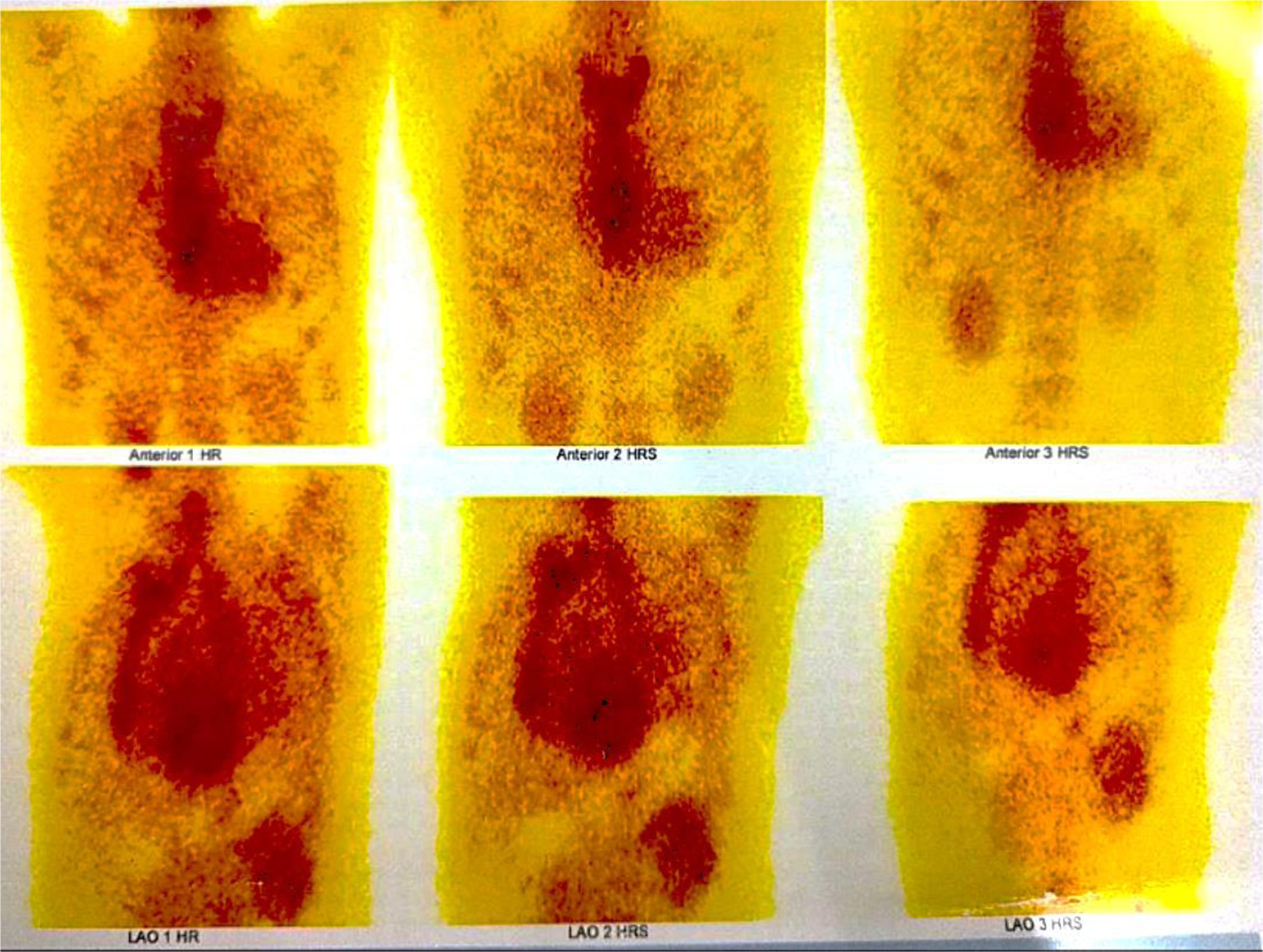
Absence of abnormal myocardial uptake in Tc-99m pyrophosphate scan.

Coronary angiography identified >70% stenosis in the mid-left anterior descending and proximal left circumflex arteries. High-resolution computed tomography of the thorax revealed smooth interlobular septal thickening, and pulmonary function tests showed restrictive patterns pre- and post-medication. Cardiac MRI with delayed contrast enhancement demonstrated diffuse global late gadolinium enhancement and biatrial dilation with increased wall signals, consistent with amyloid deposition [Figure 3]. A Tc-99m pyrophosphate scan excluded transthyretin cardiac amyloidosis [Figure 4].

Serum protein electrophoresis with immunofixation revealed elevated α-2, β-1, and β-2 globulins, high IgA (851 mg/dL; normal: 70–400 mg/dL), and elevated lambda free light chains (391.51 mg/L; normal: 5.71–26.30 mg/L) with a low kappa/lambda ratio (0.02). Fat pad and salivary gland biopsies were negative for amyloid deposition. Bone marrow biopsy showed 6–8% plasmacytosis without amyloid deposition. These findings supported a provisional diagnosis of free amyloid light-chain cardiac amyloidosis.

The patient was referred to a hematologist for targeted chemotherapy. Given the high suspicion of cardiac involvement and the evidence of inducible ischemia on stress echocardiography, percutaneous coronary revascularization was prioritized over autologous stem cell transplantation due to the increased risk of complications with coronary artery bypass grafting. Following revascularization, the patient initiated chemotherapy and was discharged with recommendations for regular hematological and cardiological follow-ups.

## Discussion

Amyloidosis is a systemic disorder marked by extracellular deposition of insoluble β-sheet fibrillar proteins, resistant to proteolytic cleavage, causing progressive organ dysfunction.^3,4^ Over 30 precursor proteins are implicated in amyloid formation, often associated with proteoglycans and serum amyloid P.^4^ Among these, cardiac involvement stands as the most critical prognostic factor, predominantly driven by two protein types: immunoglobulin-derived light chains (amyloid light-chain cardiac amyloidosis) and transthyretin (transthyretin cardiac amyloidosis). Amyloid light-chain cardiac amyloidosis remains more prevalent, with an estimated incidence of 8–12 cases per million.^4^

Historically considered rare, transthyretin cardiac amyloidosis is now being recognized more frequently, as highlighted by large autopsy series.^5^ Despite advancements in diagnostic tools, the heterogeneous presentation of cardiac amyloidosis often leads to delayed diagnoses, with dyspnea on exertion being the most common clinical symptom.^6^ Other nonspecific features, such as fatigue and low blood pressure, further complicate timely identification. The diagnostic workup demands integration of clinical findings with advanced imaging modalities, biochemical markers, and tissue biopsies. Key electrocardiographic findings include low voltage QRS complexes and left ventricular hypertrophy, while echocardiography can reveal hallmark red flags such as restrictive filling patterns and concentric hypertrophy.^7,8^

Cardiac MRI is a pivotal tool for detecting early amyloid deposition, particularly in patients with inconclusive echocardiographic findings.^9^ The capacity of cardiac MRI to identify diffuse late gadolinium enhancement provides invaluable insight into disease severity and extent of myocardial involvement.^9–11^ In this case, a comprehensive diagnostic approach confirmed amyloid light-chain cardiac amyloidosis through elevated lambda-free light chains, a low kappa/lambda ratio, and evidence of plasma cell dyscrasia from bone marrow biopsy. The absence of myocardial uptake on Tc-99m pyrophosphate scintigraphy excluded transthyretin cardiac amyloidosis, further solidifying the diagnosis.

Management of cardiac light-chain amyloidosis hinges on early detection and targeted treatment.^12–16^ For amyloid light-chain cardiac amyloidosis, chemotherapy aims to suppress the clonal plasma cell population driving amyloidogenesis.^12–16^ In our patient, concurrent coronary artery disease presented additional therapeutic challenges, necessitating revascularization before initiating chemotherapy. This multidisciplinary approach underscores the complexity of managing cardiac amyloidosis, particularly in cases with overlapping comorbidities.

Despite therapeutic advancements, cardiac amyloidosis remains associated with significant morbidity and mortality.^12–16^ Increased awareness among clinicians is essential to facilitate early diagnosis, enabling timely initiation of therapies that can stabilize disease progression and improve survival outcomes.

## Conclusion

This case highlights the diagnostic and therapeutic challenges in managing cardiac light-chain amyloidosis, particularly amyloid light-chain cardiac amyloidosis, a rare but life-threatening condition. Early detection through advanced imaging, laboratory tests, and tissue biopsies is critical for optimal management. Multidisciplinary care, integrating cardiology, hematology, and oncology expertise, is pivotal in addressing the complex needs of these patients. Continued research and heightened clinical vigilance are necessary to improve early recognition and therapeutic strategies, ultimately enhancing patient outcomes in cardiac light-chain amyloidosis.
